# *Ex vivo* modulation of the Foxo1 phosphorylation state does not lead to dysfunction of T regulatory cells

**DOI:** 10.1371/journal.pone.0173386

**Published:** 2017-03-07

**Authors:** Kristen Kelley Penberthy, Monica Weaver Buckley, Sanja Arandjelovic, Kodi Ravichandran

**Affiliations:** 1 Department of Microbiology, Immunology and Cancer Biology, University of Virginia, Charlottesville, Virginia, United States of America; 2 Center for Cell Clearance, University of Virginia, Charlottesville, Virginia, United States of America; 3 Carter Immunology Center, University of Virginia, Charlottesville, Virginia, United States of America; Baylor Institute for Immunology Research, UNITED STATES

## Abstract

Peripheral regulatory CD4^+^ T cells (Treg cells) prevent maladaptive inflammatory responses to innocuous foreign antigens. Treg cell dysfunction has been linked to many inflammatory diseases, including allergic airway inflammation. Glucocorticoids that are used to treat allergic airway inflammation and asthma are thought to work in part by promoting Treg cell differentiation; patients who are refractory to these drugs have defective induction of anti-inflammatory Treg cells. Previous observations suggest that Treg cells deficient in the transcription factor FoxO1 are pro-inflammatory, and that FoxO1 activity is regulated by its phosphorylation status and nuclear localization. Here, we asked whether altering the phosphorylation state of FoxO1 through modulation of a regulatory phosphatase might affect Treg cell function. In a mouse model of house dust mite-induced allergic airway inflammation, we observed robust recruitment of Treg cells to the lungs and lymph nodes of diseased mice, without an apparent increase in the Treg cytokine interleukin-10 in the airways. Intriguingly, expression of PP2A, a serine/threonine phosphatase linked to the regulation of FoxO1 phosphorylation, was decreased in the mediastinal lymph nodes of HDM-treated mice, mirroring the decreased PP2A expression seen in peripheral blood monocytes of glucocorticoid-resistant asthmatic patients. When we asked whether modulation of PP2A activity alters Treg cell function via treatment with the PP2A inhibitor okadaic acid, we observed increased phosphorylation of FoxO1 and decreased nuclear localization. However, dysregulation of FoxO1 did not impair Treg cell differentiation *ex vivo* or cause Treg cells to adopt a pro-inflammatory phenotype. Moreover, inhibition of PP2A activity did not affect the suppressive function of Treg cells *ex vivo*. Collectively, these data suggest that modulation of the phosphorylation state of FoxO1 via PP2A inhibition does not modify Treg cell function *ex vivo*. Our data also highlight the caveat in using *ex vivo* assays of Treg cell differentiation and function, in that while these assays are useful, they may not fully recapitulate Treg cell phenotypes that are observed *in vivo*.

## Introduction

Allergic airway inflammation is the result of maladaptive immune responses to generally innocuous foreign antigens or allergens [1–3]. One possible explanation for the development of this maladaptive inflammatory response is the functional insufficiency of the regulatory T cell (Treg) compartment [4–8]. Current evidences in the literature suggest that this functional insufficiency likely results from both decreased Treg cell numbers and Treg cell dysfunction [6,7,9–11].

Treg cells are a population of CD4^+^ T cells that are typically characterized by their expression of the transcription factor FoxP3 [12,13]. In the context of allergic airway inflammation, Treg cells are thought to suppress airway inflammation to allergens by suppressing dendritic cell activation and by producing the anti-inflammatory cytokine interleukin-10 (IL-10) [14,15]. The hypothesis that asthmatic patients have a dysfunctional Treg cell compartment is supported by the finding that the airways of patients with allergic airway inflammation have decreased IL-10 [16,17]. Furthermore, glucocorticoids, a well-established treatment for allergy, are thought to work in part by promoting Treg cell differentiation, thereby suppressing the inappropriate inflammatory response to allergens [18].

While there are multiple reports of Treg cell dysfunction in patients with allergy and asthma, how Treg cells become dysregulated in this setting is less well understood [5–7,17]. In recent years, a substantial body of work has emerged that indicates the transcription factor FoxO1 is crucial for both peripheral and thymic Treg cell differentiation and function. These studies utilized Treg and CD4^+^ T cell specific deletion of FoxO1 to demonstrate that the absence of FoxO1 impairs Treg cell differentiation and that Treg cells deficient in FoxO1 (FoxO1 was deleted after Treg cell differentiation) are pro-inflammatory and produce interferon γ (IFNγ) [19–22]. While these studies utilize elegant genetic models of gene deletion, it is possible for FoxO1 activity to become acutely dysregulated as FoxO1’s transcriptional activity is contingent on it being dephosphorylated [20,23,24]. Interestingly, the phosphatase PP2A has been shown to dephosphorylate FoxO1 in certain cell types, and patients with severe glucocorticoid-resistant asthma exhibit decreased expression of the serine-threonine phosphatase PP2A in their peripheral blood monocytes [23,25]. The significance of this finding is magnified by the observation that glucocorticoid-resistant asthmatics are also reported to have high levels of IFNγ in their airways (the same cytokine that is increased in FoxO1 deficient Treg cells) and defective induction of IL-10 producing Treg cells [17,26,27].

The transcription factor FoxO1 is crucially involved in Treg cell development and function [20,22,28]. In the absence of FoxO1, the locus for another key Treg cell transcription factor, FoxP3, fails to open, thereby preventing Treg cell differentiation [19,29,30]. Furthermore, FoxO1 suppresses the production of pro-inflammatory mediators, including IFNγ [22,24]. Under homeostatic conditions in Treg cells, FoxO1 is dephosphorylated and has an exposed nuclear localization sequence, which permits FoxO1 to carry out its transcriptional functions [23]. However, upon T cell receptor stimulation, FoxO1 is phosphorylated, which masks the nuclear localization sequence, resulting in its extrusion from the nucleus [20,23,31]. In Treg cells, rapid dephosphorylation of FoxO1 permits it to re-enter the nucleus and resume its anti-inflammatory transcriptional program [20]. Treg cells that lack FoxO1 are pro-inflammatory, and mice with FoxO1-deficient Treg cells develop systemic autoimmune disease [20]. Similarly, mice with Treg cells lacking the alpha catalytic sub-unit of PP2A (PP2Acα) exhibit profound autoimmune disease [32].

The notion that changes in PP2A expression might influence Treg cell function in allergic airway inflammation is intriguing. However, PP2A regulates diverse cellular pathways making analysis of the role of PP2A in complex biological systems challenging [33–36]. In an attempt to create an *in vitro* system to assess the role of phosphatases in Treg function, we tested the pharmacologic phosphatase inhibitor, okadaic acid. Indeed, okadaic acid caused an increase in FoxO1 phosphorylation and cytosolic sequestration in Treg cells and CD4^+^ T effector cells (Teff). However, okadaic acid did not cause Treg cells to produce IFNγ. Furthermore, okadaic acid treatment did not recapitulate the findings from the recent study that utilized an *in vivo* model of PP2A catalytic sub-unit deletion in Treg cells [32]. Specifically, okadaic acid treated Treg cells did not produce IL-17 nor did they demonstrate impaired suppression of CD4^+^ effector proliferation. Collectively, these data suggest that *ex vivo* treatment of Treg cells with okadaic acid and the resultant modulation of PP2A activity and FoxO1 phosphorylation are not sufficient to modulate the functional activity of Treg cells. These findings also suggest a caution for others attempting to utilize *ex vivo* assays to assesses the effects of altering PP2A activity / FoxO1 phosphorylation status in Treg cells.

## Materials and methods

### Ethics statement

All animal experiments conducted in this study were carried out in strict accordance with protocols approved by the University of Virginia Animal Care and Use Committee (Protocol number: 2992). All experiments followed the recommendations in the Guide for the Care and Use of Laboratory Animals of the National Institutes of Health (OLAW/NIH, 2002) and followed the requirements of the Animal Welfare Act (Public Law 91–579). All efforts were made to minimize animal suffering including the use of anesthesia (isoflurane delivered at 5% for induction and 3% for maintenance in oxygen in a precision vaporizer) for the administration of house dust mite (HDM). Mice were monitored daily by vivarium staff and were euthanized at the experimental endpoint in a carbon dioxide chamber followed by confirmation via cervical dislocation. These methods are consistent with the recommendations of the Panel on Euthanasia and approved by the UVA Animal Care and Use Committee.

### Animals and primary cell culture

Mice used in airway inflammation studies were C57Bl/6J purchased from Jackson Laboratories. For primary CD4^+^ T cell cultures, total CD4^+^ T cells were isolated from the lymph nodes and spleens of either C57Bl6/J mice or FoxP3-EGFP mice (Stock 016961 from Jackson Laboratories) by negative magnetic selection with the MACS CD4^+^ T cell isolation kit (Miltenyi). Cells were cultured in RPMI (Cell Gro) supplemented with 10% FBS, 1% PSQ, 1% non-essential amino acids, 1% Sodium Pyruvate, and 10mM HEPES (Gibco). For long-term assays, cell culture plates were coated with antibodies to CD3 and CD28 (eBiosciences—clones 17A2 and 37.51, respectively) to promote activation and proliferation. Short-term stimulation assays were performed using soluble CD3 and CD28, cross-linked with anti-hamster IgG (Jackson Immunolabs, 107-005-142). Cultures were treated with calyculin A (Calbiochem), okadaic acid (Sigma) or LB100 (Selleckchem).

### Mouse model of allergic airway inflammation

House dust mite extract was purchased from Greer laboratories (XPB82D3A2.5) and reconstituted in PBS such that the DerP1 concentration was 0.87 mg/mL. Mice were anesthetized by isoflurane and 17.4 μg of DerP1 was administered intranasally in a 50 μL volume on days 0, 2, and 4 for antigen priming. HDM was again administered on days 10, 12 and 14 as an antigen challenge. 50μL of PBS was administered to control mice at all time points. On day 15, mice were euthanized and the severity of airway inflammation was analyzed. Bronchoalveolar spaces were lavaged with 1 mL of PBS for analysis of T cell infiltration (T effectors: CD4^+^CD25^+^FoxP3^−^, T regulatory cells: CD4^+^CD25^+^FoxP3^+^) and eosinophilia (CD45^+^Siglec F^+^CD11b^+^CD11c^−^). The following antibodies were used at a dilution of 1:100 for flow cytometric analysis: CD4 (eBioscience GK1.5), CD25 (eBioscience PC61.5), FoxP3 (eBioscience FJK-16s), CD45 (eBioscience RA3-6B2), Siglec F (BD E50-2440), CD11b (M1/70 eBioscience), CD11c (eBioscience N148). Right lung lobes were analyzed by histology and left lung lobes were analyzed by flow cytometry to assess tissue infiltrate. Mediastinal lymph nodes were collected and analyzed by flow cytometry and RT-PCR. Cell populations were quantified by flow cytometry via Spherotech counting beads (Spherotech ACBP-50-10).

### RT-PCR

RNA was isolated with the RNeasy RNA isolation kit (Qiagen) and cDNA was prepared using the Quantitect kit (Qiagen). Expression of the PP2A catalytic subunit was assessed by RT-PCR with a Taqman probe (Mm00479816_m1, Thermo Fisher). HPRT probe (Mm00446968_m1, Thermo Fisher) was used to assess housekeeping gene expression for normalization.

### Flow cytometry

Single cell suspensions were generated from tissue by passing through a 70μm cell strainer (lungs were first digested and minced in 0.6 mg/mL type-2 collagenase (Worthington Biochemical) for 1 hour at 37°C to promote tissue breakdown). Cell suspensions were incubated with anti-CD16/32 antibodies (1:200 dilution; eBioscience clone 93) to block Fc interactions. Subsequent extracellular antibody labeling was performed on ice in PBS supplemented with 0.5% BSA (Roche). Viability staining was performed with 7-AAD (1:1000 dilution; BD Bioscience) for live-cell analysis, or an amine reactive fixable viability dye (1:1000 dilution; FVD eFluor780 eBioscience) when cells were to be fixed and permeabilized. For intracellular labeling, cells were fixed and permeabilized with either the FoxP3 fixation buffer kit or IC fixation buffer kit from eBioscience, according to manufacturer recommendations. Intracellular antibody labeling was performed for 30 minutes on ice in permeabilization buffer (eBioscience). All flow cytometry was performed on BD FACS Canto I and Canto II. Flow cytometric data was analyzed in Flow Jo software version 887 (FlowJo LLC).

### Protein isolation and immunoblotting

Whole cell lysates were prepared in RIPA buffer supplemented with a protease inhibitor cocktail (Calbiochem), 10mM NaF and 1mM NaVO_3_ (Sigma). Nuclear and cytoplasmic extraction was performed with the NE-Per kit (Pierce). Lysates were analyzed by SDS-PAGE and immunoblotting. Antibody binding was detected by chemiluminescence on either film or a ChemiDoc Touch (Bio-Rad). Immunoblot band density analysis and normalization was performed using ImageJ for analysis of film or Image Lab (Bio-Rad) for analysis of ChemiDoc Touch images. The following primary antibodies were purchased from Cell Signaling Technologies and used at 1:1000 dilutions unless otherwise indicated: phospho-FoxO1 (Thr24) (#9464), FoxO1 (clone C29H4—#2880), HDAC1 (#2062), phospho-Akt (ser473) (#4060S) Akt (1:2000; #9272). The antibody to β-actin was purchased from Sigma and used at 1:50,000 (clone AC-15, #A3854).

### Phosphatase assay

Total CD4^+^ T cells were stimulated with 5μg/mL anti-CD3 and 1μg/mL anti-CD28 for 10 minutes to elicit FoxO1 phosphorylation. CD4^+^ T cells were lysed in IP buffer (50mM Tris pH 7.6, 150mM NaCl, 1% Triton X-100, protease inhibitor cocktail (Calbiochem), 10mM NaF, 1mM NaVO_3_) and incubated with anti-FoxO1 (clone C29H4—#2880). Antibody bound FoxO1 was immunoprecipitated with Protein A conjugated sepharose beads. Beads were washed in the IP buffer without NaF and NaVO_3_ to ensure irreversible phosphatase inhibitors would not inhibit the subsequent phosphatase reaction. Beads were then re-suspended in phosphatase assay buffer (50mM MOPS pH 7.5, 100mM NaCl, 10mM MgSO_4_, 1mM MnCl_2_, 1mM DTT, 1mM EDTA, 0.1% β mercaptoethanol, protease inhibitor cocktail (Calbiochem)) with purified PP2A +/- 100 nM okadaic acid. Purified (tested and validated) PP2A was generously provided by Dr. David Brautigan at the University of Virginia [37]. Phosphatase reactions were incubated for 1 hour at 30°C. Following incubation, beads were re-suspended in Laemmli buffer and boiled. Laemmli buffer was then loaded on an SDS-PAGE gel for immunoblot analysis.

### T cell proliferation and suppression assays

Total CD4^+^ T cells were isolated from lymph nodes and spleens of naïve mice by negative magnetic selection. The positive fraction from the spleen was treated with mitomycin C (Sigma) to inhibit proliferation and used as antigen presenting cells. Treg cells were further isolated from the total CD4^+^ T cell population by positive selection for CD25 expression. CD4^+^ Teff cells constituted the negative fraction of this CD25 selection. Teff cells were labeled with 5μM Cell Trace Violet (Thermo Fisher). APCs and Teff cells were co-cultured at a 1:1 ratio with 0.15 μg/mL soluble anti-CD3 (eBiosciences 17A2) to elicit proliferation. For suppression assays, Treg cells were added at the indicated ratios (Treg: Teff). Proliferation of Teff cells was analyzed by dye dilution on a BD Canto II flow cytometer after 96 hours of culture.

### T regulatory cell differentiation

Total CD4^+^ T cells were isolated from lymph nodes of FoxP3 EGFP mice and cultured *ex vivo* in media supplemented with 20 nM retinoic acid (sigma), 5 or 10 ng/mL TGFβ (R&D systems) and recombinant IL-2 (R&D systems) at either 100 or 250U/mL. When assessing the effects of okadaic acid on Treg cell differentiation, the lower concentrations of TGFβ and IL-2 were used. The higher concentrations of TGFβ and IL-2 were used to generate maximum numbers of Tregs. Cells were plated on dishes coated with anti-CD3 and anti-CD28 (clones 17A2 and 37.51 respectively; eBiosciences). Cultures were incubated for 72 hours and flow cytometry was used to assess FoxP3 induction and CD25 expression. To assess cytokine production, GolgiPlug (BD Biosciences) was added to the culture for 5 hours prior to staining for IFNγ (clone XMG1.2; eBioscience) and IL-17 staining (clone TC11-18H10; BD).

## Results

### Allergen-induced airway inflammation increases local Treg cell populations

**Fig 1:**
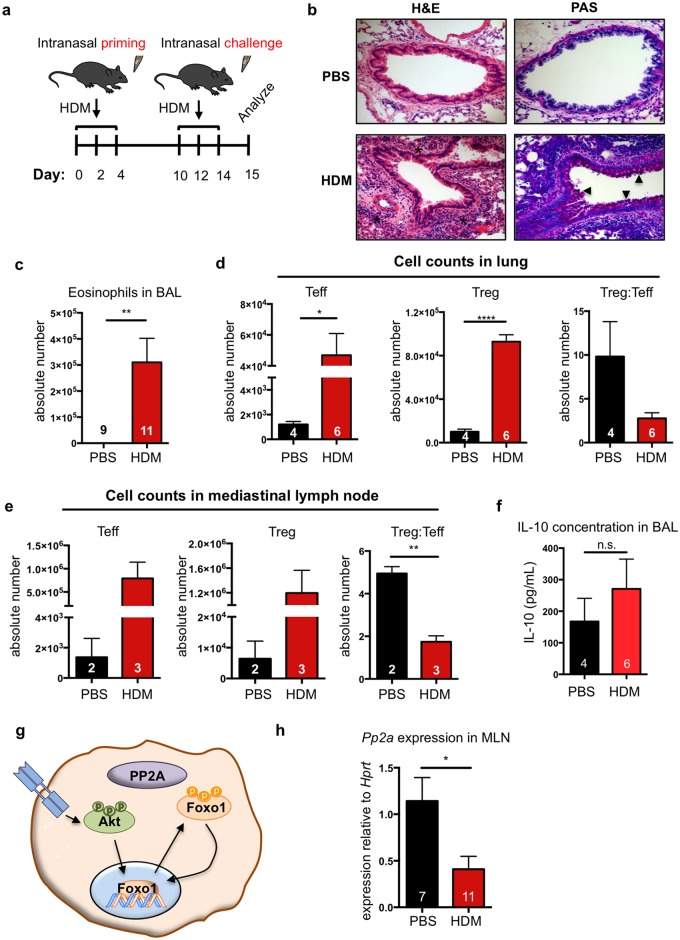
HDM potently induces allergic airway inflammation despite significant T regulatory cell recruitment. **(a)** Allergic airway inflammation was induced with three separate priming and challenge doses of house dust mite (HDM) over 14 days. Disease severity was assessed at day 15. **(b)** Histological assessment of inflammation in lung sections of representative HDM-treated and PBS-treated mice. * in H&E images indicates inflammatory infiltrate and arrowheads in PAS images indicate mucus. **(c)** Eosinophils were quantified in the bronchoalveolar lavage (BAL) by flow cytometry (CD45^+^Cd11b^+^SiglecF^+^CD11c^−^). Treg (CD4^+^CD25^+^FoxP3^+^) Teff (CD4^+^CD25^+^FoxP3^−^) populations in the lung parenchyma **(d)** and mediastinal lymph nodes (MLN) **(e)** were quantified by flow cytometry. **(f)** IL-10 concentration in BAL was quantified by ELISA. **(g)** Schematic of PP2A-FoxO1 axis. **(h)** PP2Acα sub-unit transcript levels were measured by RT-PCR. Transcript levels in HDM-treated mice were normalized to PBS-treated controls. Numbers in the bars of **c, d, e, f** and **h** represent the number of animals analyzed. Significance in all panels was determined by two-tailed students’ T-test: *<0.05; **<0.01; ****<0.0001.

Patients with severe, glucocorticoid-resistant allergic asthma are reported to have a dysfunctional Treg cell compartment [6,17]. Treg cell dysfunction is thought to underlie the defective tolerogenic response that leads to the development of allergy [38]. To test potential Treg cell dysfunction, we used a murine model of severe allergic airway inflammation. In this model, the common allergen house dust mite (HDM) is delivered to mice intranasally, which mimics the typical route of exposure to airborne allergens. We treated mice with three intranasal administrations of HDM on days 0, 2 and 4 to prime the immune system and subsequently challenged the mice intranasally with HDM on days 10, 12 and 14 (Fig 1a). The inflammatory response was assessed on day 15.

We initially assessed whether this model caused severe airway disease and whether Treg cells were recruited to the site of inflammation. HDM-treated mice exhibited classic signs of allergic airway inflammation. First, hematoxylin and eosin (H&E) staining of lung sections revealed marked cellular infiltration around the airways of HDM-treated mice (indicated with black asterisks) compared to mice that received the saline (PBS) vehicle control (Fig 1b, left panels). Second, Periodic acid-Schiff (PAS) stain, which detects mucus production, showed dramatically increased mucus production (marked by black arrowheads) in HDM-treated mice relative to PBS-treated controls (Fig 1b, right panels). Additionally, analysis of bronchoalveolar lavage (BAL) fluid showed severe eosinophilia in HDM-treated animals relative to PBS-treated controls (Fig 1c).

Analysis of CD4^+^ T cell populations of lungs from HDM-treated mice showed a significant increase in the number of Teff cells (CD4^+^CD25^+^FoxP3^−^) and Treg cells (CD4^+^CD25^+^FoxP3^+^), compared to PBS controls (Fig 1d). It should be noted that the ratio of Treg cells to Teff cells trended toward a decrease in HDM-treated mice relative to PBS controls (Fig 1d). We performed a similar analysis of CD4^+^ T cell populations in the lung-draining, mediastinal lymph nodes (MLN), and observed a similar trend with more Teff cells and Treg cells in HDM-treated mice (Fig 1e). Additionally, the ratio of Treg cells to Teff cells was significantly decreased in the MLN of HDM-treated mice (Fig 1e). These data confirmed that this model of airway inflammation recapitulated key characteristics of allergic airway inflammation.

### Mice with HDM-induced inflammation do not exhibit increased IL-10

Treg cells are major producers of IL-10, and IL-10 levels inversely correlate with airway inflammation [11,16,39,40]. Since Treg populations in the lung increased with HDM-induced airway inflammation, we asked whether IL-10 levels in the BAL fluid had a concomitant increase. HDM-treated mice did not exhibit increased IL-10 levels in their BAL fluid, despite the increase in Treg numbers (Fig 1f). This suggested a potential Treg dysfunction in this airway inflammation model, which we further explored, as detailed below.

### Mediastinal lymph nodes from mice with allergic airway inflammation have decreased expression of the phosphatase PP2A

FoxO1 transcriptional activity is tightly regulated by a series of phosphorylation and dephosphorylation reactions [41,42]. In a resting CD4^+^ T cell, FoxO1 exists in a dephosphorylated state and localizes to the nucleus [20]. However, upon T cell receptor (TCR) stimulation, Akt phosphorylates FoxO1, which masks a nuclear localization sequence leading to cytosolic sequestration (Fig 1g) [1–3,20,43]. FoxO1 transcriptional activity is critical to proper Treg anti-inflammatory function, with Treg cells rapidly dephosphorylating FoxO1 to maintain their suppressive activity. Treg cells that lack FoxO1 upregulate expression of the pro-inflammatory cytokine interferon γ (IFNγ), the production of which is linked to severe, glucocorticoid-resistant asthma in humans [4–8,26,27]. We considered the possibility that FoxO1 might be dysregulated under conditions of allergic airway inflammation, thereby rendering Treg cells pro-inflammatory.

While the mediator of FoxO1 dephosphorylation in Treg cells was previously unexplored, PP2A has been shown to dephosphorylate FoxO1 in the hematopoietic cell line FL5.12 [6,7,9–11,23]. Since PP2A expression is down regulated in the peripheral blood monocytes of patients with severe, glucocorticoid-resistant asthma [12,13,25], the same group reported to have increased IFNγ production, we hypothesized that decreased PP2A expression might promote dysregulation of FoxO1, subsequently causing aberrant IFNγ production in Tregs cells and potentially greater overall dysfunction. Expression of the PP2A catalytic sub-unit α (PP2Acα) was decreased in the MLN of HDM-treated mice (Fig 1h) [14,15,25]. This prompted us to explore whether alterations in PP2A activity in Treg cells might perturb FoxO1 activity and thereby Treg cell function.

### Phosphatase inhibitors increase FoxO1 phosphorylation and nuclear localization in total CD4^+^ Cells

Following TCR activation, FoxO1 is rapidly phosphorylated and extruded from the nucleus. To assess whether phosphatase inhibition would influence the kinetics and extent of FoxO1 phosphorylation following TCR stimulation, we utilized the pharmacologic inhibitor calyculin A, a fast-acting, broad phosphatase inhibitor [16,17,44]. Indeed, treatment of total CD4^+^ T cells with calyculin A prior to TCR stimulation led to increased phosphorylation of FoxO1 (S1a and S1b Fig). Calyculin A treatment also led to an apparent increase in the molecular weight of FoxO1 and decreased FoxO1 expression. This appeared to result from calyculin A-dependent ubiquitylation and proteasomal degradation of FoxO1, as the decrease in FoxO1 expression, but not the apparent size change, was abrogated by proteasomal inhibition with lactacystin (S1c Fig).

**Fig 2:**
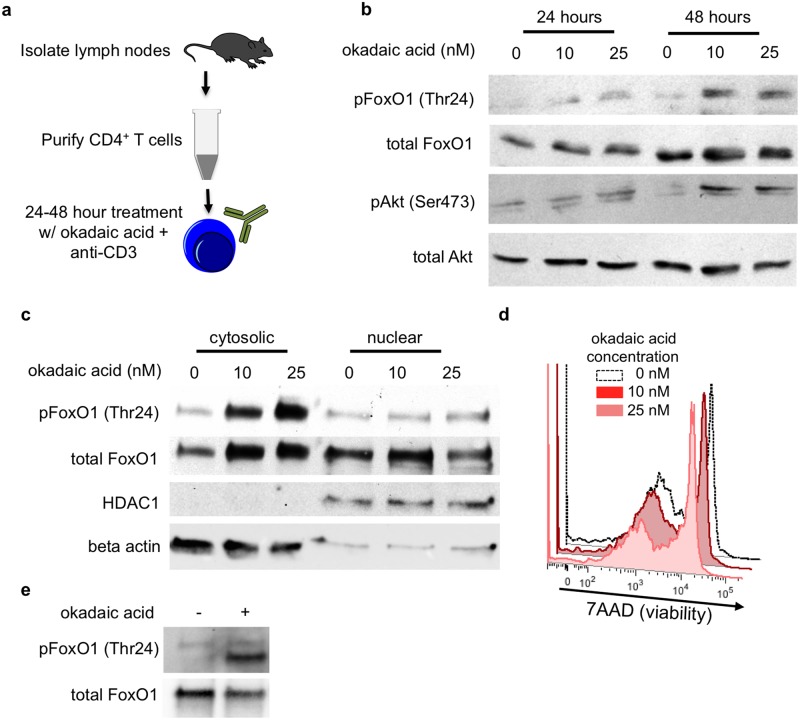
PP2A inhibition increases FoxO1 phosphorylation and cytosolic localization in CD4^+^ T cells. **(a)** Primary CD4^+^ T cells were negatively selected from the lymph nodes of 6–8 week old mice by magnetic cell sorting. CD4^+^ T cells were treated with the indicated dosages of okadaic acid for up to 48 hours in cultures supplemented with 1μg/mL anti-CD3. **(b)** Western blot analysis of FoxO1 phosphorylation at Thr24 and Akt at Ser 473. **(c)** Western blot analysis of FoxO1 localization in fractionated CD4 cells. Beta-actin and HDAC were used as cytoplasmic and nuclear controls respectively. **(d)** Viability of CD4^+^ T cells in culture after 48 hours with the indicated concentrations of okadaic acid. **(e)** Western blot analysis of in vitro phosphatase assay. Immunoprecipitated FoxO1 was treated with PP2A +/- 100nM okadaic acid. **b, c and d** are representative data from 3 individual experiments; **e** is representative of 2 experiments.

Calyculin A has the benefit of rapid activity in culture however, it exhibits near-equal activity against PP1 and PP2A [18,44]. Therefore, we tested the phosphatase inhibitor okadaic acid, as this exhibits greater preference for PP2A than for PP1 (IC_50_ for PP2A 0.1–0.3 nM, IC_50_ for PP1 15–50 nM) [5–7,17,44]. Although okadaic acid can affect PP4, the expression of PP4 is lower than PP2A and PP1 in Treg cells [19–22,45]. Total CD4^+^ T cells were isolated from the lymph nodes of naïve mice and treated *ex vivo* with increasing concentrations of okadaic acid for 48 hours (Fig 2a). We observed that concentrations of 10 and 25 nM were sufficient to induce Akt and FoxO1 phosphorylation in total CD4^+^ T cells (Fig 2b). Furthermore, okadaic acid treatment of CD4^+^ T cells led to increased cytosolic localization of FoxO1 (Fig 2c). For future experiments, we elected to use 10 nM okadaic acid as this concentration fell below the IC_50_ for PP1 (15–50 nM) and 25 nM okadaic acid had some effects (albeit minimal) on cell viability after 48 hours (Fig 2d).

Since okadaic acid also increases Akt phosphorylation, it could be enhancing FoxO1 phosphorylation indirectly by enhancing Akt activity. To demonstrate that PP2A directly mediates the dephosphorylation of FoxO1, we assayed PP2A activity against purified FoxO1 *in vitro*. First, total CD4^+^ T cells were stimulated with anti-CD3 and anti-CD28 to increase FoxO1 phosphorylation. These cells were subsequently lysed and FoxO1 was purified by immunoprecipitation. Immunoprecipitated FoxO1 was then treated with purified PP2A +/- 100nM okadaic acid. Treatment of FoxO1 with PP2A eliminated FoxO1 phosphorylation. This effect on phosphorylation was reversed by treatment with okadaic acid (Fig 2e). Collectively, these data suggest that phosphatase inhibition with okadaic acid promoted FoxO1 phosphorylation and subsequent cytosolic localization, likely through its effect on PP2A.

Though okadaic acid at the concentration used predominantly inhibits PP2A, it has the potential for off-target effects. Therefore, we tested LB100, a second PP2A inhibitor. LB100 is a derivative of norcantharidin with high specificity for PP2A (IC_50_≈0.4μM) over PP1 (IC_50_≈80μM) [20,23,24,46]. To test the effect of LB100 on FoxO1 phosphorylation, we treated total CD4^+^ T cells for 2 hours in the presence of 0, 0.25μM, or 2.5μM LB100. Following 2 hours of treatment, we collected whole cell lysates and assayed FoxO1 phosphorylation. We observed a dose-dependent augmentation of FoxO1 phosphorylation in the presence of LB100 (S2 Fig). These data suggest that the changes in FoxO1 phosphorylation are not due to off-target effects of okadaic acid.

### Okadaic acid promotes FoxO1 phosphorylation in Treg cells

**Fig 3:**
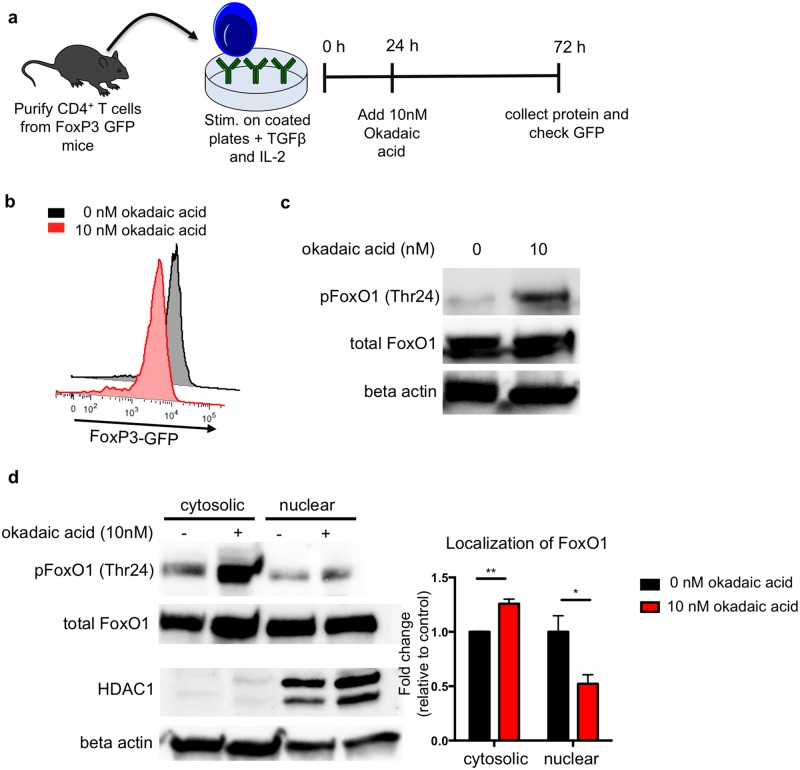
Okadaic acid increases phosphorylation and cytosolic localization of FoxO1 in T regulatory cells. **(a)** Primary CD4^+^ T cells were isolated by negative magnetic selection from the lymph nodes of FoxP3-GFP mice. Cells were cultured on plates coated with anti-CD3 and anti-CD28 and treated with TGFβ to initiate Treg skewing. Okadaic acid was added after 24 hours in culture. After 72 hours in culture, cells were removed from TCR stimulus for 2 hours prior to protein isolation. **(b)** Treg differentiation was assessed at the end of the assay by GFP expression. **(c)** Representative western blot of FoxO1 phosphorylation at Thr24 in Treg cells. Data are representative of 3 independent experiments. **(d)** Representative western blot of FoxO1 in fractionated Treg cells. Beta actin and HDAC were used as cytosolic and nuclear controls respectively. Bar graph shows summary localization data from 3 experiments as measured by band density and normalized to cytosolic control.

We then tested whether okadaic acid was capable of promoting FoxO1 phosphorylation in Treg cells. Since the Treg cell population in a naïve mouse is approximately 10% of the total CD4^+^ T cell population, we differentiated Treg cells from naïve CD4^+^ T cells *ex vivo* to have enough cells for protein isolation and immunoblotting. Naïve CD4^+^ T cells were isolated from the lymph nodes of FoxP3-GFP mice and stimulated with anti-CD3 and anti-CD28 in the presence of TGFβ to elicit FoxP3 expression. As FoxO1 transcriptional activity is critical for the early induction of FoxP3, we waited 24 hours before adding okadaic acid or vehicle control (DMSO) to the culture (Fig 3a) [23,25,29]. After 72 hours of culture, we confirmed the induction of FoxP3 expression by assessing GFP signal. Furthermore, we determined that treatment with okadaic acid did not diminish FoxP3 induction as measured by GFP signal (Fig 3b). Analysis of the cell lysates from these Treg cells revealed that okadaic acid treated Treg cells had increased FoxO1 phosphorylation (Fig 3c) and increased cytosolic localization of FoxO1 (Fig 3d). These data suggest that okadaic acid promotes FoxO1 phosphorylation and causes its cytosolic sequestration in Treg cells.

### Okadaic acid does not impair Treg differentiation ex vivo

**Fig 4:**
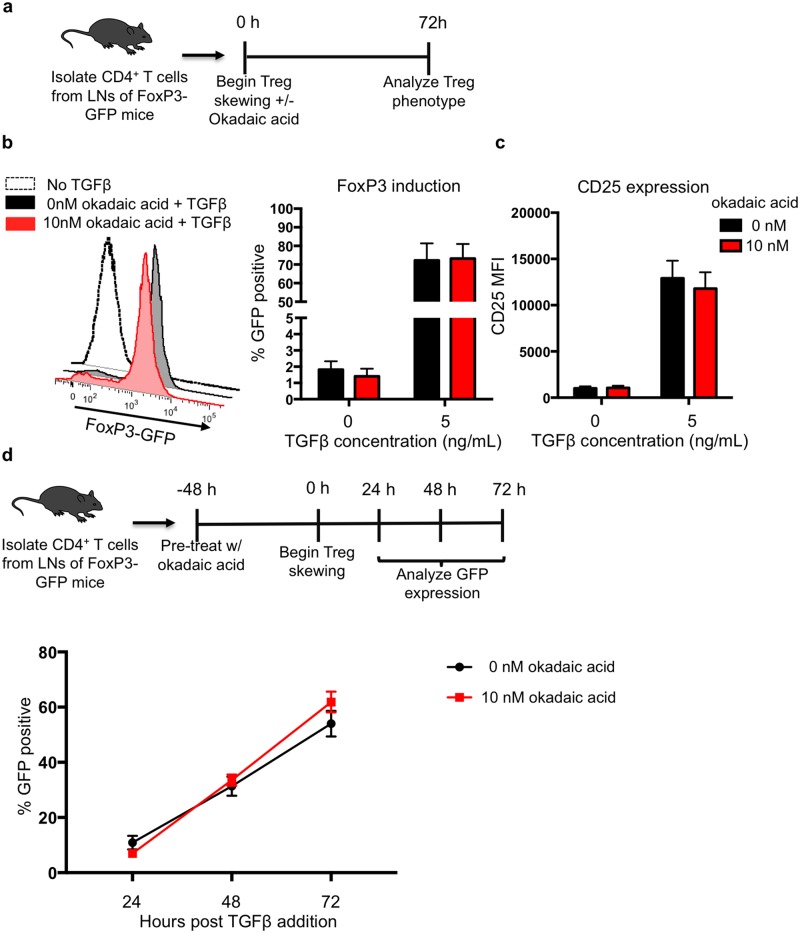
Okadaic acid does not impair T regulatory cell differentiation in vitro. **(a)** Naïve CD4 cells were isolated from the lymph nodes of FoxP3-GFP mice and differentiated in to Tregs for 72 hours. **(b)** GFP and **(c)** CD25 expression were assessed by flow cytometry after 72 hours of Treg skewing. Data were compiled from 3 independent experiments with a total n = 6 mice per group. **(d)** Primary CD4 cells were isolated from the lymph nodes of FoxP3 GFP mice and pre-treated with okadaic acid for 48 hours prior to Treg differentiation. GFP expression was assessed by flow cytometry at 24, 48 and 72 hours after the initiation of skewing. Data were compiled from 3 independent experiments with n = 6 mice per group. Statistical analysis of **b** and **c** was performed using a two-tailed students’ T-test–comparison of vehicle control and okadaic acid treated conditions: FoxP3 p = 0.7; CD25 p = 0.12. Statistical analysis of **e** was performed by 2-way ANOVA–p-interaction = 0.19.

To test whether okadaic acid-induced changes in FoxO1 localization had downstream effects on Treg cell physiology, we chose to assess whether okadaic acid impaired Treg cell differentiation when added at the start of the skewing. Since FoxO1 transcriptional activity is essential for the induction of FoxP3 expression [17,19,26,27,29], we used assays of *ex vivo* Treg cell differentiation to assess whether okadaic acid impaired FoxP3 induction. Naïve CD4^+^ T cells were isolated from FoxP3-GFP mice and treated with either okadaic acid or vehicle in a Treg cell differentiation assay. In these assays, TGFβ, IL-2, TCR stimulus and okadaic acid were added concomitantly (Fig 4a). Interestingly, when we assessed GFP expression after 72 hours, we observed that okadaic acid did not impair FoxP3 induction (Fig 4b). Furthermore, okadaic acid did not affect CD25 expression on either the GFP positive (Treg cells) or GFP negative cells from the culture (Fig 4c).

One possible explanation for the lack of an effect of okadaic acid on Treg cell differentiation is that okadaic acid requires 48 hours to achieve maximum enhancement of FoxO1 phosphorylation in culture (Fig 2b). Since FoxO1 is essential for opening the FoxP3 locus (but not maintaining its open state) [20,22,28,47], we hypothesized that pre-treatment of CD4^+^ T cells with okadaic acid may be necessary to observe okadaic acid-induced defects in Treg cell differentiation. Therefore, naïve CD4^+^ T cells from FoxP3-GFP mice were treated with okadaic acid and CD3 for 48 hours prior to the addition of TGFβ, IL-2, and CD28. Once the Treg skewing stimuli were applied, we assessed FoxP3 expression by measuring GFP signal (Fig 4d). Again, okadaic acid pre-treatment did not diminish FoxP3 induction (Fig 4e). These data indicate that okadaic acid does not influence Treg differentiation *in vitro*, despite increasing cytosolic localization of FoxO1.

### Okadaic acid does not affect the phenotype of ex vivo differentiated Treg cells

**Fig 5:**
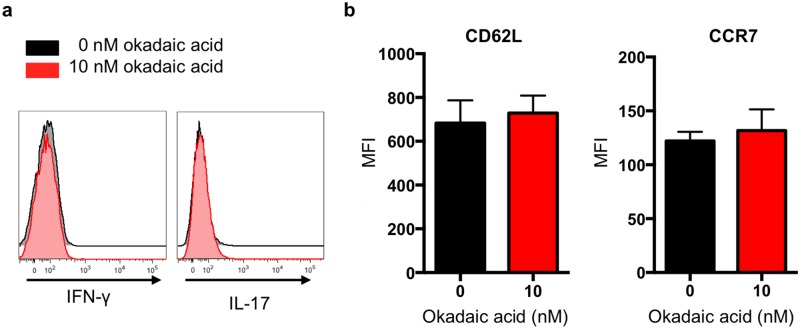
Okadaic acid does not induce a pro-inflammatory phenotype or affect lymph node homing signatures in Treg cells. Primary CD4^+^ T cells were differentiated into Tregs in the presence of okadaic acid for 72 hours. **(a)** After differentiation, IFNγ and IL-17 production and **(b)** CCR7 and CD62L expression were measured by flow cytometry. Flow panels of cytokine expression are representative data from 3 independent experiments. Surface marker expression was compiled from 2 independent experiments with n = 3 mice total. Data in **b** was analyzed by non-parametric students’ T-test: CD62L p = 0.5; CCR7 p = 0.77.

Mice that lack FoxO1 in Treg cells (FoxP3-cre FoxO1^fl/fl^) mice exhibit severe autoimmune disease that is reminiscent of the disease found in FoxP3-deficient *scurfy* mice [19,20,29,30,48]. However, the lack of FoxO1 does not affect the number of CD4^+^FoxP3^+^ cells in these animals. Instead, the Treg cells from these mice adopt a pro-inflammatory phenotype and produce the pro-inflammatory cytokine IFNγ [20,22,24]. Therefore, we hypothesized that Treg cells differentiated in the presence of okadaic acid would exhibit increased IFNγ production. However, when we assessed IFNγ expression by flow cytometry we observed that okadaic acid did not cause IFNγ up regulation in Treg cells (Fig 5a).

It had also been reported that ‘graded’ changes in FoxO1 activity influenced lymph node homing signatures on Treg cells [23,24]. FoxO1 promotes expression of the lymph node homing molecules CD62L and CCR7 [20,23,31,49]. In Treg cells from mice that express increasing levels of constitutively active FoxO1, the expression of these lymph node homing molecules similarly increases [20,24]. Since okadaic acid treatment did not cause complete cytosolic localization of FoxO1 (Fig 3d), we hypothesized that we might only see effects associated with ‘graded’ changes in FoxO1 localization. However, when we assessed CCR7 and CD62L expression on Treg cells differentiated in the presence of okadaic acid, we did not observe changes in the expression of these molecules (Fig 5b).

During the course of our studies, a study utilizing an elegant *in vivo* genetic deletion model for the assessment of PP2A function in Treg cells demonstrated that PP2A was essential for Treg cell function [20,32]. The researchers deleted the dominant catalytic sub-unit of PP2A in Treg cells (FoxP3-cre PP2Acα^fl/fl^) and observed that these Treg cells produced inflammatory cytokines, including IFNγ and IL-17. In light of these findings, we also evaluated IL-17 production in Treg cells that were differentiated in the presence of okadaic acid. Surprisingly, we observed that okadaic acid did not affect IL-17 production by Treg cells (Fig 5a).

### Okadaic acid treatment of Treg cells does not influence Treg suppression ex vivo

**Fig 6:**
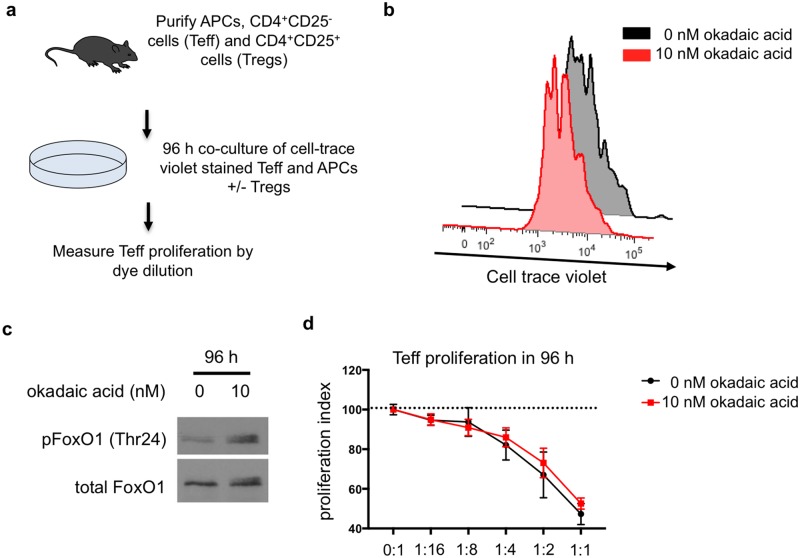
Okadaic acid does not impair T regulatory cell mediated suppression of CD4 effector cells. **(a)** Teff and Tregs were isolated from the lymph nodes and spleen of FoxP3-GFP mice. Cells were co-cultured at indicated ratios in the presence of antigen presenting cells and 0.15 μg/mL anti-CD3 for 96 hours +/- okadaic acid. **(b)** CD4^+^ T cell proliferation in the presence of okadaic acid was measured by cell-trace violet dye dilution. Data is representative of 4 independent experiments. **(c)** Western blot of FoxO1 phosphorylation at Thr24 in CD4^+^ T cells treated with okadaic acid for 96 hours. Data is representative of 2 independent experiments. **(d)** Proliferation index of Teff cells in the presence of increasing numbers of Tregs was assessed by cell-trace violet dye dilution. Data is compiled from 4 independent experiments–statistical analysis was performed by 2-way ANOVA; p-interaction = 0.44.

Treg cells that lack the alpha catalytic sub-unit of PP2A have a decreased suppressive capacity [20,32]. Therefore, we assessed whether okadaic acid would influence the suppressive capacity of Treg cells *ex vivo*. We utilized a standard suppression assay in which co-cultured Treg cells and Teff cells were treated with okadaic acid (Fig 6a). First, we assessed whether okadaic acid influenced Teff proliferation *ex vivo* and observed that okadaic acid treated Teff cells proliferated to the same extent as vehicle treated controls (Fig 6b). In addition, we confirmed that okadaic acid retained its activity for the duration of the assay as okadaic acid treated CD4^+^ T cells still exhibited increased FoxO1 phosphorylation at 96 hours (Fig 6c). We then went on to assess the effect of okadaic acid on Treg cell suppression of Teff proliferation. Interestingly, okadaic acid did not affect Treg cell suppressive capacity in these assays (Fig 6d). Collectively, these data indicate that okadaic acid, despite inhibiting PP2A activity and promoting FoxO1 phosphorylation, does not influence Treg cell phenotype and function *in vitro*.

## Discussion

Allergic airway inflammation and asthma occur due to a breakdown in host immune tolerance to harmless foreign antigens or allergens. In normal individuals, allergens elicit an anti-inflammatory response. However, in patients and experimental animal models with severe allergic airway inflammation and airway hyper-reactivity, a maladaptive inflammatory response develops [1–3,33–36]. Treg cell dysfunction has been proposed as a possible reason for the breakdown in tolerance and the subsequent inflammatory response [5–7,32]. However, it is unclear how Treg physiology could be subtly altered in a specific inflammatory setting without leading to a broader defect. The purpose of this study was to investigate whether the phenomenon of decreased PP2A expression in asthmatics might be linked to Treg cell dysfunction.

In a mouse model of house dust mite-induced allergic airway inflammation, we observed severe airway inflammation characterized by eosinophilia, Teff cell infiltration, and increased mucus production. These animals also had high numbers of Treg cells in their lungs and mediastinal lymph nodes, yet had no significant increase in IL-10 levels in their airways. As IL-10 is a cytokine often associated with peripheral Treg cells, we hypothesized that these mice could be exhibiting Treg cell dysfunction. Interestingly, these inflamed mice exhibited decreased PP2A expression, a feature previously observed in glucocorticoid resistant asthmatics [6,17,25]. This was of particular interest as PP2A was previously shown to regulate activity of the critical Treg cell transcription factor, FoxO1 [23,38].

FoxO1 expression is essential for Treg cell differentiation as well as the Treg cell anti-inflammatory phenotype [11,16,19–21,39,40]. Deletion of FoxO1 in Treg cells causes them to adopt a pro-inflammatory phenotype and produce IFNγ. Interestingly, increased levels of IFNγ are observed in patients with severe, glucocorticoid-resistant asthma—the same category of patients that have decreased PP2A expression. We hypothesized that FoxO1 function could become perturbed under conditions with decreased PP2A expression, as PP2A promotes FoxO1 nuclear localization. To determine whether changes in PP2A activity could alter Treg cell phenotypes, we utilized the PP2A inhibitor okadaic acid in a series of *in vitro* assays to test Treg cell differentiation and function.

Surprisingly, we did not observe any perturbations of the Treg cell phenotype in the presence of okadaic acid. Okadaic acid treated Treg cells did not produce inflammatory cytokines, such as IL-17 or IFNγ, both of which have been reported in Treg cells deficient in PP2A and FoxO1 [20,32]. Additionally, we observed that the suppressive function of Treg cells on Teff cell proliferation was unaffected by okadaic acid-mediated PP2A inhibition, in contrast to Treg cells lacking PP2Acα [32]. From these data we conclude that *in vitro* administration of okadaic acid is an unsuitable experimental system for further dissecting the role of phosphatases in Treg function.

While *in vivo* studies, including genetic deletion of phosphatases, provide valuable information, complementary *ex vivo* assays are critical for mechanistic studies, and for assessing potential therapeutic options prior to testing in an organismal context. Therefore, while we carefully analyzed multiple parameters to ensure that okadaic acid blocked phosphatases and affected FoxO1 phosphorylation status, okadaic acid did not recapitulate the functional deficiencies of Treg cells that have been reported in Treg cells that lack FoxO1 and Treg cells that lack the catalytic subunit of PP2A. This could be due to at least two possibilities. First, the loss of FoxO1 or PP2A achieved genetically occurs throughout the ‘life’ of the Treg cells prior to their analysis. In our *ex vivo* assays, this is done via acute inhibition of PP2A, and whether the acute versus chronic loss of PP2A function differentially affects Treg phenotype remains to be seen. Second, the function of Treg cells is likely complex and precisely how Treg cells regulate Teff cells *in vivo* is still incompletely understood. Therefore, it is possible that acute inhibition of PP2A activity and subsequent alteration of FoxO1 phosphorylation might lead to subtle changes in Treg cell function that cannot be observed in *ex vivo* systems. Given the *in vivo* findings from other groups regarding the importance of FoxO1 and PP2A in Treg cells, our work suggests that caution should be used in interpreting the *ex vivo* assays of Treg cell function as they correlate to Treg cell functionality *in vivo*.

While our *in vitro* systems were unable to recapitulate the recent *in vivo* observations regarding the importance of PP2A in Treg cell function [32], it is an intriguing possibility that the decrease in PP2A expression observed in the mediatstinal lymph nodes of mice with allergic airway inflammation, and in the peripheral blood monocytes of patients with glucocorticoid resistant asthma, might be associated with Treg cell dysfunction. We hope that further use of genetic models, ideally with temporal manipulation of gene expression, might elucidate how PP2A and FoxO1 function and regulation might influence various acquired inflammatory states.

## Supporting information

S1 Fig Calyculin A increases FoxO1 phosphorylation and ubiquitylation. **(a)** Total CD4^+^ T cells were isolated from the lymph nodes of mice by magnetic cell sorting and treated with calyculin A or DMSO (vehicle control) for 30 minutes prior to stimulation with soluble, cross-linked anti-CD3 and anti-CD28. **(b)** Cells were stimulated for the indicated amount of time and then lysed for analysis of FoxO1 phosphorylation at Thr24. Arrows point to higher molecular weight forms of FoxO1. **(c)** Following isolation, CD4^+^ T cells were treated with Calyculin A and/or the proteasomal inhibitor lactacystin. Data are representative of 2 independent experiments.
(TIF)

S2 Fig Phosphatase inhibitor LB100 enhances FoxO1 phosphorylation. Primary CD4^+^ T cells were isolated from the lymph nodes of WT mice and serum starved for 2 hours in the presence of 0 μM, 0.25 μM and 2.5 μM LB100. After 2 hours, whole cell lysates were collected and FoxO1 phosphorylation was analyzed by western blot. Data are representative of 3 independent experiments.
(TIF)
