# Supplementary figures and images for: Ex vivo modulation of the Foxo1 phosphorylation state does not lead to dysfunction of T regulatory cells
Source: PLoS One. 2017 Mar 7;12(3):e0173386. doi: 10.1371/journal.pone.0173386 (PMC5340387; doi:10.1371/journal.pone.0173386)

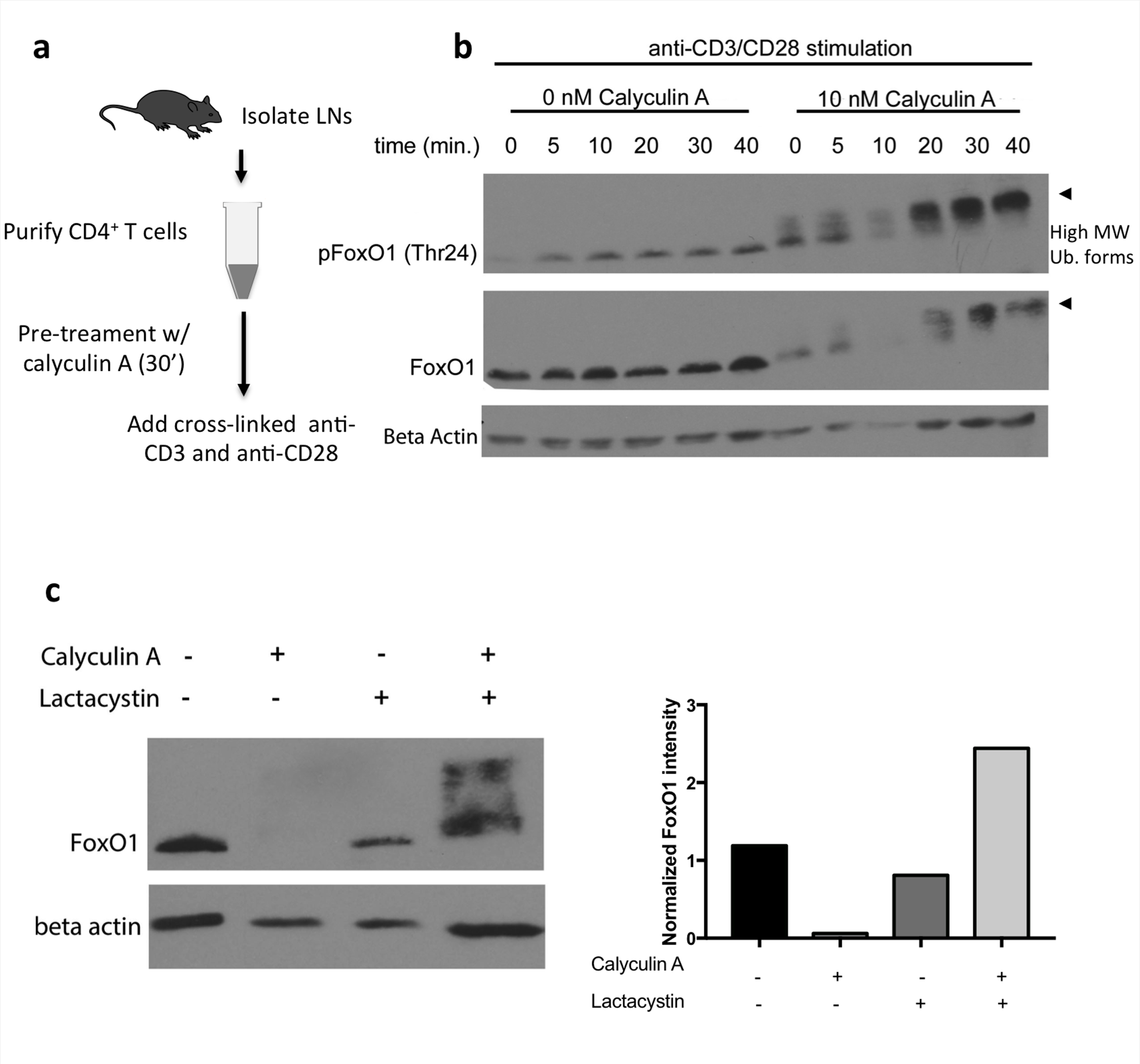

Supplement: S1 Fig — (a) Total CD4+ T cells were isolated from the lymph nodes of mice by magnetic cell sorting and treated with calyculin A or DMSO (vehicle control) for 30 minutes prior to stimulation with soluble, cross-linked anti-CD3 and anti-CD28. (b) Cells were stimulated for the indicated amount of time and then lysed for analysis of FoxO1 phosphorylation at Thr24. Arrows point to higher molecular weight forms of FoxO1. (c) Following isolation, CD4+ T cells were treated with Calyculin A and/or the proteasomal inhibitor lactacystin. Data are representative of 2 independent experiments. (TIF) [file pone.0173386.s001.tif]

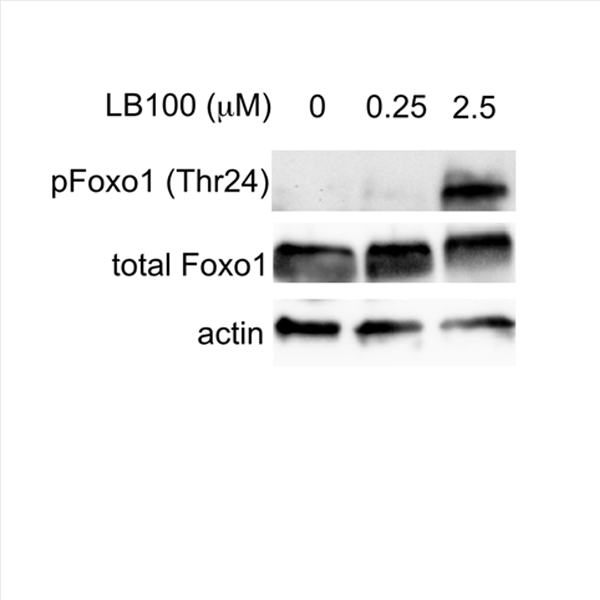

Supplement: S2 Fig — Primary CD4+ T cells were isolated from the lymph nodes of WT mice and serum starved for 2 hours in the presence of 0 μM, 0.25 μM and 2.5 μM LB100. After 2 hours, whole cell lysates were collected and FoxO1 phosphorylation was analyzed by western blot. Data are representative of 3 independent experiments. (TIF) [file pone.0173386.s002.tif]
